# Foul Wells as a Cause of Typhoid Fever

**Published:** 1873-10

**Authors:** D. Colvin

**Affiliations:** Clyde, N. Y.


### Miscellaneous.

### Foul Wells as a Cause of Typhoid Fever.

By D. Colvin, M. D., Clyde, N. Y.

Fearing that there yet may be some who doubt the drinking of impure water to be a fruitful cause for the development of typhoid fever, I deem it to be my duty to contribute to the columns of your journal some recent experience I have had, and which is, I think, sufficient proof of the fact.

I was recently summoned many miles into the country for the purpose of visiting the son of a farmer, fifteen years of age. I found him with the usual symptoms of typhoid fever.

While in attendance, and within four days, I was asked to see another son, aged ten, in an adjoining room.

He was then confined to the bed the greater part of the time, having had the symptoms attending the prodromic period for about a week. Not yet suspecting the real cause, I made no particular inquiry relative to the condition of the premises except so far as related to the cellar. I found that to be, with a slight exception, free from any poisonous matter.

Within three days I was again asked to see still another child, a little girl aged seven, who was suffering almost precisely as were the other two.

I then began an investigation relative to the house-drain, and found, so far as I then deemed it necessary to examine, nothing which I considered of sufficient cause to produce the particular illness.

But when within three or four days my attention was again called to another child, aged twelve years and four months, I concluded there must be some cause, aside from contagion, which could be discovered. I might add that the children composing the family numbering eight in all, and that upon summoning the balance, and instituting the necessary inquiries, I found that two of the remaining four were complaining of "not feeling well." "Some diarrhœa, with chilly feelings and headache."

I then desired the drain, which received the slops of the house, to be thoroughly opened. It ran in the direction of the well, the water of which was used for the family's general use, although it passed it to the right some four feet. Upon inspection, the following day, I found that there was a perfect communication between the drain and well at a point directly opposite the well, so that all matters thrown into the drain passed directly into the well. And found all lhe surfaces of the drain beyond this opening perfectly dry.

I might state that this occurred at "a very dry time," and the water in the well, as I was informed, was much lower than it had been in years. Its use was at once abandoned, but its effects did not cease until the two children who were then slightly ailing, and the mother, took to the bed, and all passed safely through a severe trial of the disease, save the mother, who succumbed to it in six days from the time she went to bed.

The only ones of the family who escaped the terrible scourge were the father and oldest daughter; unless the youngest child, which was still nursing when the mother was seized, was amenable to it (which I very much doubt).

I will not extend this paper by any fdrther remarks, as it will now occupy too much of your valuable space.—*Medical Record.*

